# Theaflavin-3,3′-Digallate Targets Pin1 to Suppress Hepatocellular Carcinoma Malignant Proliferation Through Modulation of MAPK and PI3K/AKT Signaling Pathways In Vitro

**DOI:** 10.3390/biom16040583

**Published:** 2026-04-14

**Authors:** Shaoli Lv, Wenli Jiang, Jingyi Liu, Jiaxin Tao, Hui Zhong, Huaqing He, Xinling Liao, Jiayang Xie, Xiyuan Ouyang, Wang Wang

**Affiliations:** 1 Key Laboratory of Infection and Immunity, Health Commission of Jiangxi Province, School of Basic Medicine, Nanchang Medical College, Nanchang 330052, China; lvshaoli@ncmc.edu.cn (S.L.); jiangwenli@ncmc.edu.cn (W.J.); liujingyi11@ncmc.edu.cn (J.L.); 2022020100171@ncmc.edu.cn (J.T.); 202202010026@ncmc.edu.cn (H.Z.); hehuaqing@ncmc.edu.cn (H.H.); liaoxinling@ncmc.edu.cn (X.L.); xiejiayang@ncmc.edu.cn (J.X.); ouyangxiyuan@ncmc.edu.cn (X.O.); 2 Jiangxi Provincial Education Department Key Laboratory for the Application of Key Technologies in Drug Screening for Inflammatory Diseases and Phlegm Syndrome, Nanchang 330052, China; 3 The Jiangxi Province Key Laboratory of Hematologic Diseases, Nanchang 330052, China

**Keywords:** Pin1, TF3, hepatocellular carcinoma, PI3K/AKT, MAPK

## Abstract

Theaflavin-3,3′-digallate (TF3), a flavan-3-ol derivative found in black tea, exhibits anti-tumor activity, but its mechanism of action in hepatocellular carcinoma (HCC) remains to be elucidated. Here we systematically delineate how TF3 targets Pin1 to suppress HCC through an integrated approach combining computational simulations, enzyme assay and cell-based assays. TF3 spontaneously occupies the active site of Pin1 with a docking score of −8.9 kcal/mol, inhibiting its PPIase activity (IC_50_ = 60.33 μmol/L) and yielding a binding constant (*K_a_*) of 3.1 × 10^5^ mol/L. Drug affinity responsive target stability (DARTS) assays further corroborated that TF3 directly engages Pin1 within HCC cells. Functionally, TF3 potently suppressed the viability of HepG2, SK-Hep-1 and Huh-7 cells in both dose- and time-dependent manners (IC_50_ = 61.22, 14.09 and 69.85 μmol/L at 24 h, respectively), and exhibited a modest selectivity window against the viability of L02 and THLE-2 cells (IC_50_ = 133.43 and 90.29 μmol/L at 24 h, respectively). In addition, TF3 triggers mitochondrial-mediated apoptosis, evidenced by ROS accumulation, loss of mitochondrial membrane potential, an elevated Bax/Bcl-2 ratio, cytochrome c release and enhanced PARP cleavage, and induces G_2_/M phase arrest. It also robustly inhibits HCC cell proliferation, invasion and migration, coinciding with downregulation of proteins governing cell cycle progression and invasive behavior. Transcriptome profiling coupled with enrichment analysis discovered that TF3 treatment differentially regulated 5009 genes, which were prominently enriched in pathways linked to apoptosis, cell cycle control, MAPK and PI3K/AKT signaling pathways. Western blotting analysis revealed that TF3 selectively suppresses phosphorylation of p38 and the PI3K/AKT cascade, activating JNK phosphorylation. In summary, our findings indicate that TF3 suppresses HCC proliferation by targeting Pin1, with attendant modulation of the MAPK and PI3K/AKT pathways, thereby presenting a potential candidate for targeted HCC therapy.

## 1. Introduction

Hepatocellular carcinoma (HCC) is one of the leading causes of cancer-related deaths worldwide, and its occurrence and development are closely related to the abnormal activation of multiple signaling pathways [1,2]. Although targeted therapy and immunotherapy have made certain progress in recent years, the overall prognosis of HCC patients remains unsatisfactory, facing challenges such as drug resistance, recurrence, and treatment-related toxic and side effects [3,4,5]. Therefore, discovering new lead compounds that are highly efficient and low-toxicity from natural products and deeply clarifying their mechanisms of action remain one of the important strategies in the current research and development of anti-HCC drugs [6,7].

Tea, as a widely popular drink, contains a variety of bioactive components in its extracts [8,9]. Among them, theaflavins are a class of key polyphenolic compounds formed during the fermentation of black tea, which have been proven to have antioxidant, anti-inflammatory and potential anti-cancer activities [10,11]. Theaflavin-3,3′-digallate (TF3) is a highly active member of the theaflavin family [12,13]. Recent studies have shown that TF3 has demonstrated effects such as inhibiting proliferation, inducing apoptosis and blocking the cell cycle in various tumor models [14,15,16]. However, the specific molecular targets of its anti-HCC and the downstream signal network regulation mechanism have not been fully clarified, which seriously restricts the clinical transformation and precision drug design based on TF3.

Prolyl isomerase Pin1, as a unique phosphorylation-dependent prolyl cis-trans isomerase, acts as a key molecular switch in cellular signal transduction [17]. It regulates the stability and function of cell cycle, apoptosis, metabolism and metastasis-related proteins by specifically recognizing phosphorylated serine/threonine-proline (pSer/Thr-Pro) motifs and catalyzing the conformational transformation of substrate proteins [18,19]. In various malignant tumors including hepatocellular carcinoma, Pin1 often shows abnormally high expression [20,21]. Its activity can drive tumorigenesis and development by stabilizing proto-oncoproteins such as c-Myc and β-catenin or interfering with the functions of tumor suppressor proteins such as p53 [22,23,24]. It is particularly worth noting that Pin1 can directly or indirectly regulate multiple nodes in core signaling pathways such as MAPK and PI3K/Akt, and thus is a highly promising molecular target in the treatment of liver cancer [25,26,27].

**Figure 1:**
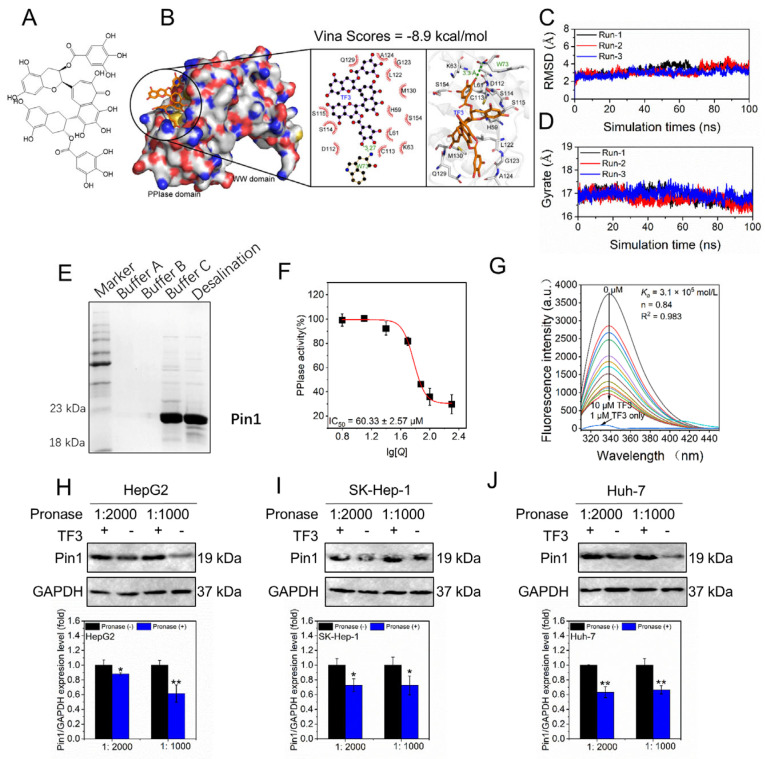
TF3 targets Pin1 and inhibits its PPIase activity. (**A**). The chemical structure of TF3. (**B**). Molecular docking predicts the binding mode of TF3 with the active center of Pin1. (**C**,**D**). Molecular dynamics simulation was used to analyze the RMSD and Rg values of the Pin1-TF3 complex. (**E**). SDS-PAGE was used to detect the purity of recombinant Pin1. (**F**). The inhibitory effect of TF3 on the activity of Pin1 PPIase enzyme. (**G**). Measurement of the binding constant (*K_a_*) and stoichiometric ratio (*n*) of TF3 and Pin1 using fluorescence titration. (**H**–**J**). DARTS experiment verified the direct binding of TF3 to Pin1 in liver cancer cells. * 0.01 < *p* < 0.05, significant difference, ** *p* < 0.01, highly significant difference.

In recent years, research on small molecule inhibitors targeting Pin1 has continued to make progress [28]. In addition to the early-discovered natural inhibitors such as naphthoquinone, more compounds with optimized potency or selectivity (such as API-1 [29], API-32 [30], Sulfopin [31],) and various natural products (such as all-trans retinoic acid [26], epigallocatechin-3-gallate [32], rhein [33], ellagic acid [34], ginsenoside Rh2 [35]) have also been reported to have Pin1 inhibitory activity. However, existing inhibitors still generally face challenges such as insufficient selectivity, limited efficacy or poor pharmacokinetic properties [36]. Particularly crucial is that Pin1 regulates downstream signaling pathways through its extensive substrate network [37]. However, the mechanism by which its inhibitors systematically affect key signal transduction within liver cancer cells and ultimately determine cell fate has not yet been fully elucidated. Theaflavin-3,3′-digallate is a characteristic polyphenolic compound in black tea (Figure 1A) [38]. Although it has shown anti-cancer potential, whether it directly targets Pin1 and its specific mechanism of action in liver cancer still needs to be further revealed.

Based on the above background, this study aims to systematically explore whether TF3 mediates its anti-liver cancer activity by targeting Pin1. Firstly, the direct binding of TF3 to Pin1 and its inhibitory effect on enzyme activity were verified by comprehensively applying molecular docking and molecular dynamics simulation, PPIase enzyme activity inhibition, fluorescence titration and drug affinity target stability (DARTS) experiments. Subsequently, in multiple hepatoma cell lines, the effects of TF3 on malignant phenotypes such as cell viability, apoptosis, cell cycle progression, clone formation, migration and invasion were evaluated, and its role in the stability of Pin1 protein was detected. Furthermore, through transcriptome sequencing (RNA-seq) and bioinformatics analysis, combined with Western blotting verification of key signaling proteins, the regulatory effect of TF3 on signaling pathways such as MAPK and PI3K/Akt/mTOR was clarified. This study reveals the molecular mechanism by which TF3, as a novel Pin1 inhibitor, inhibits the growth of liver cancer by targeting Pin1 and disturbing the downstream signaling network, providing an experimental basis for the in-depth study of its anti-liver cancer effect.

## 2. Materials and Methods

### 2.1. Materials

L02, HepG2, SK-Hep-1, and Huh-7 cells were purchased from Suzhou Haixing Biotechnology Co., Ltd. (Suzhou, China). THLE-2 cells and their specialized culture medium were purchased from Zhejiang Baidi Biotechnology Co., Ltd. (Hangzhou, China). DMEM, MEM medium, fetal bovine serum, 0.25% trypsin digestion solution, and penicillin-streptomycin solution were purchased from Wuhan Procell Biotechnology Co., Ltd. (Wuhan, China). TF3 standard was purchased from Chengdu Medson Technology Co., Ltd. (Chengdu, China). Suc-AEPF-pNA was purchased from Nanjing Peptide Biotechnology Co., Ltd. (Nanjing, China). Pronase was purchased from Shanghai Yuanye Biotechnology Co., Ltd. (Shanghai, China). CCK-8 assay kit, BCA assay kit, apoptosis and necrosis detection kit (Hoechst 33342/PI), Annexin V-FITC apoptosis detection kit, cell cycle and apoptosis detection kit, mitochondrial membrane potential detection kit (JC-1), reactive oxygen species detection Kit (DCFH-DA) and the antibodies of β-actin, GAPDH, cyclin D1, cyclin B1, CDK2, CDK4, CDK6, N-cadherin, vimentin, MMP2, MMP9, horseradish peroxidase-labeled goat anti-rabbit IgG (H + L), and horseradish peroxidase-labeled goat anti-mouse IgG (H + L) were purchased from Shanghai Beyotime Biotechnology Co., Ltd. (Shanghai, China). The antibodies of Pin1, Bax, Bcl-2, PARP, Cyto-c, ERK, p-ERK, p38, p-p38, JNK, p-JNK, PI3K, p-PI3K, AKT, p-AKT, mTOR, p-mTOR, p70S6K and p-p70S6K were purchased from Wuhan Proteintech Biotechnology Co., Ltd. (Wuhan, China).

### 2.2. Methods

#### 2.2.1. Computational Simulations

This study investigated the interaction between Pin1 and TF3 through molecular docking using AutoDock Vina v1.0 [39]. First, the crystal structure of Pin1 (PDB ID: 3OOB) was downloaded from the RCSB PDB database [32]. Preprocessing was conducted using AutoDock Tools software v1.5.2, including removal of crystalline molecules, addition of polar hydrogen atoms, and assignment of Kollman charges. Next, the 3D structure of TF3 (ZINC ID: ZINC150338650) was obtained from the ZINC15 database [40]. Both Pin1 and TF3 were subsequently converted into PDBQT format, the input standard for AutoDock Vina. To define the docking search space, a grid box measuring 40 Å × 40 Å × 40 Å was positioned with its center at coordinates 35 Å × 24 Å × 26 Å, ensuring coverage of the PPIase domain of Pin1. Finally, visualization and interaction analysis were carried out in PyMOL v1.0 and LigPlot^+^ v2.3.1 software [41,42].

To assess the solution stability of the Pin1-TF3 complex, we conducted molecular dynamics simulations using GROMACS v2022 software based on the optimal binding conformation obtained from molecular docking [43]. First, the topology and parameter files of TF3 were generated using antechamber under the GAFF parameter set. Subsequently, the complex was centered in a cubic box within TIP3P water molecules, and neutralized by replacing an appropriate number of solvent molecules with Na^+^ and Cl^−^ ions. Energy minimization was subsequently performed to eliminate steric clashes and unfavorable contacts, followed by stepwise equilibration under NVT and NPT ensembles. The production run was carried out for 100 ns in the NPT ensemble, with bond constraints enforced via the LINCS algorithm and long-range electrostatics treated using the particle mesh Ewald (PME) method. Finally, the trajectory was processed with GROMACS built-in tools to compute the RMSD and radius of gyration of the complex.

#### 2.2.2. Prokaryotic Expression and Purification of Pin1

BL21(DE3) pLysS cells transformed with the pET19b-Pin1 plasmid were cultured in LB medium containing ampicillin [44]. Protein expression was induced with 0.5 mM IPTG at 20 °C for 12 h. The bacterial cells were collected by centrifugation, resuspended in pre-cooled Buffer A (20 mM Tris-HCl, 500 mM NaCl, 20 mM imidazole, pH 8.0, containing 1 mM PMSF), ultrasonically lysed in an ice bath, and the supernatant was obtained by centrifugation. The clarified lysate was then loaded onto a column packed with His-tag Purification Resin. The column was washed sequentially with Buffer B (20 mM Tris-HCl, 500 mM NaCl, 10 mM imidazole, pH 8.0), and bound protein was eluted with Buffer C (20 mM Tris-HCl, 500 mM NaCl, 100 mM imidazole, pH 8.0). The elution peak was collected and subsequently processed on a Shanghai Union-Biotech UEV25L protein purification system (Yonglian Biotechnology Co., Ltd., Shanghai, China) equipped with a G-25 desalting column, where the buffer was exchanged to Buffer D (20 mM Tris-HCl, 150 mM NaCl, pH 7.5) to remove imidazole and excess salts. Protein purity and concentration were assessed by SDS-PAGE and Bradford assay, respectively.

#### 2.2.3. PPIase Enzyme Activity Assay

The peptidyl-prolyl cis-trans isomerase (PPIase) activity of Pin1 was assessed using a standard protease-coupled assay [32]. In brief, the different concentrations (0–200 μM) of TF3 were preincubated with 100 nmol/L Pin1 at 4 °C for 10 min. Then, α-chymotrypsin was added to a final concentration of 0.1 mg/mL and mixed rapidly. The substrate peptide Suc-AEPF-pNA was immediately added to a final concentration of 100 μmol/L to initiate the enzymatic reaction. The absorbance values at 395 nm were monitored kinetically using the HBS-ScanX microplate reader (Nanjing DeTie Laboratory Equipment Co., Ltd., Nanjing, China). The inhibition rate of TF3 on Pin1 activity was calculated based on the reaction rate.

#### 2.2.4. Fluorescence Spectroscopy

Pin1 protein was first diluted to a working concentration of 1 μM in a quartz cuvette, and its intrinsic fluorescence was recorded on a FL970 fluorescence spectrophotometer (Shanghai Techcomp Instrument Ltd., Shanghai, China) [44]. The excitation wavelength was fixed at 295 nm, with both excitation and emission slit widths set to 5 nm. Emission spectra were collected across the 310–450 nm range at 298 K. Using a micro-syringe, small aliquots of TF3 solution were titrated into the cuvette, yielding final concentrations from 0 to 10 μM. Subsequently, the complete emission spectrum was collected. Ultimately, by analyzing the fluorescence intensity quenching value of Pin1 at the maximum emission wavelength (approximately 340 nm), the binding constant (*K_a_*) and stoichiometric ratio (*n*) of between Pin1 and TF3 were calculated using the modified *Stern*-*Volmer* equation [45].

#### 2.2.5. Western Blotting

After protein quantification using the BCA assay, sample concentrations were first normalized. Proteins were then mixed with 5× loading buffer, heat-denatured, and resolved by SDS-PAGE. Wet transfer onto PVDF membranes was carried out at 200–300 mA for approximately 2–3 h. After transfer, the membrane was blocked with 5% non-fat milk or 5% BSA at room temperature for 1 h, followed by overnight incubation with the primary antibody at 4 °C. The next day, membranes were washed three times in TTBS (10 min each) and subsequently probed with HRP-conjugated secondary antibodies for 1 h at room temperature. After another round of TTBS washes, the blots were evenly covered with ECL substrate. Chemiluminescent signals were captured using the 4600SF imaging system (Tanon Science & Technology Co., Ltd., Shanghai, China), and band intensities were quantified with Image J v1.48 software.

#### 2.2.6. Drug Affinity Responsive Targeting Stability (DARTS) Assay

First, cell lysates were divided into two groups and incubated with TF3 or DMSO at 4 °C for 1 h. Subsequently, both groups were subjected to enzymatic digestion with pronase at dilutions of 1:1000 and 1:2000, incubated at room temperature for 1 h. The proteolytic reaction was immediately terminated by adding pre-chilled PMSF, and all samples were denatured by boiling. Finally, Western blotting was carried out to detect the proteins of interest.

#### 2.2.7. Cell Culture

Five human hepatocyte cell lines (L02, THLE-2, HepG2, Huh-7, and SK-Hep-1) were employed in this study. All cells were maintained in a humidified incubator at 37 °C and 5% CO_2_, with routine handling performed in a biosafety cabinet. L02, HepG2, Huh-7 and SK-Hep-1 cells were cultured in high-glucose DMEM medium supplemented with 10% fetal bovine serum and 1% penicillin and streptomycin solution. THLE-2 cells required a specialized complete medium for cultivation. Cells were passaged via trypsin digestion as needed and kept in logarithmic growth phase for subsequent experimental use.

#### 2.2.8. CCK-8 Assay

To evaluate the effect of theaflavin TF3 on the viability of liver cancer cells and normal hepatocytes, the CCK-8 assay was performed. TF3 was dissolved in DMSO, and the final concentration of DMSO in all treatment groups was maintained below 0.1%. First, cells were seeded into 96-well plates at optimized densities and cultured overnight. Subsequently, cells were exposed to a graded series of TF3 concentrations ranging from 0 to 200 μM. After the addition of drug-containing medium, the cells were incubated for 24, 48, or 72 h. At each time point, 10 μL of CCK-8 reagent was added to each well, and the plates were incubated in the dark for another 1–4 h. Finally, absorbance was measured at 450 nm using the HBS-ScanX microplate reader (Nanjing DeTie Laboratory Equipment Co., Ltd., Nanjing, China) and cell viability was determined accordingly.

#### 2.2.9. Hoechst 33342/PI, DCFH-DA, and JC-1 Staining Assays

To evaluate apoptosis, cells were stained using the Hoechst 33342/PI apoptosis and necrosis detection kit (Beyotime Biotechnology Co., Ltd., Shanghai, China). First, cells in the logarithmic growth phase were seeded at an appropriate density in 24-well plates and cultured overnight. After TF3 treatment and further overnight incubation, the cells were washed with PBS. Sequentially, Hoechst 33342 and PI staining solutions were added to a final concentration of 10 μg/mL each, and the mixture was incubated in the dark at room temperature for 15 min. After staining was completed, the staining solution was discarded, the cells were washed with PBS, basic culture medium was added, and the cells were immediately observed under a Leica DMIL LED Fluo inverted fluorescence microscope (Leica Microsystems, Wetzlar, Germany).

The effect of TF3 on reactive oxygen species in cells was detected using the reactive oxygen species detection kit (DCFH-DA, Beyotime Biotechnology Co., Ltd., Shanghai, China). First, cells in the logarithmic growth phase were seeded into 24-well plates at an appropriate density and cultured overnight. Subsequently, TF3 was added to treat the cells, and they were cultured overnight, then washed with PBS. A DCFH-DA probe with a final concentration of 10 μM was added and incubated in the dark for 30 min. After staining was completed, the staining solution was discarded, the cells were washed with PBS, basic culture medium was added, and the cells were immediately observed under a Leica DMIL LED Fluo inverted fluorescence microscope (Leica Microsystems, Wetzlar, Germany).

The effect of TF3 on the mitochondrial membrane potential of cells was detected using the mitochondrial membrane potential detection kit (JC-1, Beyotime Biotechnology Co., Ltd., Shanghai, China). First, cells in the logarithmic growth phase were seeded into 24-well plates at an appropriate density and cultured overnight. Subsequently, the cells were treated with TF3, cultured overnight, and washed with PBS. 1× JC-1 probe was added and incubated in the dark for 30 min. After staining was completed, the staining solution was discarded, the cells were washed with PBS, basic culture medium was added, and the cells were immediately observed under a Leica DMIL LED Fluo inverted fluorescence microscope (Leica Microsystems, Wetzlar, Germany).

#### 2.2.10. Flow Cytometry Analysis of Apoptosis and Cell Cycle

The effect of TF3 on apoptosis was detected using the Annexin V-FITC/PI apoptosis detection kit (Beyotime Biotechnology Co., Ltd., Shanghai, China). First, cells from each group treated with TF3 were collected and washed twice with pre-cooled PBS. Subsequently, approximately 1 × 10^6^ cells were resuspended in 100 μL Binding Buffer, followed by the addition of 5 μL Annexin V-FITC staining solution and 10 μL PI staining solution. After gentle mixing, the mixture was incubated at room temperature in the dark for 15 min. After the reaction was completed, 400 μL of PBS buffer was immediately added, and apoptosis was detected by Beckman CytoFLEX flow cytometry (Beckman Coulter Life Sciences, Suzhou, China).

The effect of TF3 on the cell cycle was detected using the apoptosis and cell cycle detection kit (Beyotime Biotechnology Co., Ltd., Shanghai, China). First, the treated cells were collected and washed twice with pre-cooled PBS. Subsequently, the cells were fixed at 4 °C with 70% ice-ethanol for 2 h. After fixation, the cells were centrifuged to discard ethanol and resuspended with PBS for washing. Then approximately 1 × 10^6^ cells were added to the PI staining working solution containing RNase A and incubated in the dark at 37 °C for 30 min. Next, transfer the upper sample tube and immediately use the Beckman CytoFLEX flow cytometer for detection (Beckman Coulter Life Sciences, Suzhou, China).

#### 2.2.11. Colony Formation

First, cells in the logarithmic growth phase were collected, digested with trypsin and counted. Then, they were diluted in a gradient with complete culture medium and seeded into six-well plates at a low density of approximately 500 to 1000 cells per well, and gently shaken to disperse evenly. The cells were placed in a 37 °C incubator with 5% CO_2_ for routine culture. After about a week, fresh culture medium was replaced and the culture continued until visible cell clones appeared to the naked eye. Subsequently, an appropriate amount of TF3 was added to treat the cells. The culture medium was changed every 3 to 4 days. After the culture was completed, the culture medium was discarded, and the cells were carefully washed with PBS. Then, the cells were fixed with 4% paraformaldehyde for 30 min and stained with 0.1% crystal violet for another 30 min. Finally, excess dye was washed off slowly with running water, dried at room temperature, analyzed with Image J v1.48 software, and the clone formation rate was calculated.

#### 2.2.12. Transwell Chamber Assay

First, the matrix gel (Matrigel) was diluted with pre-cooled serum-free medium at a ratio of 1:8, 50 μL was added to each small chamber and evenly spread. The plates were incubated at 37 °C for 4 h to polymerize. Logarithmic growth phase cells were digested and collected, resuspended in serum-free medium and the concentration was adjusted to 1 × 10^5^ cells/mL. 500 μL of complete medium containing 10% FBS was added to the lower chamber and 200 μL of cell suspension treated with different concentrations of TF3 was added to the upper chamber. The culture plate was incubated in the incubator for 24 h. Then the small chamber was taken out, the un-invaded cells in the upper inner layer were gently wiped off with a cotton swab, fixed with 4% paraformaldehyde for 30 min, and stained with 0.1% crystal violet for 30 min. After gentle rinsing with PBS, photos were taken under an inverted microscope of Ningbo Yongxin NIB620 (Ningbo, China) and analyzed using Image J v1.48 software to quantify the number of invaded cells.

#### 2.2.13. Scratch Wound Assay

First, cells in the logarithmic growth phase were seeded into a 6-well plate. Uniform horizontal lines were drawn on the back of the plate bottom in advance using a sterile Marker pen as observation references. When the cells had grown to nearly 100% confluence, a scratch was made by steadily and uniformly sliding the tip of a 200 µL sterile pipette across the cell layer, perpendicular to the reference line. Then, detached cells were gently washed away with PBS, and the medium was replaced with 1% FBS medium containing various concentrations of TF3. The intersection points of the scratch and reference lines were immediately marked under an inverted microscope. The same fields were photographed at 0 and 24 h. Finally, wound areas at each time point were measured using Image J v1.48 software, and cell migration rate was calculated as (area of 0 h–area of 24 h)/area of 0 h × 100%.

#### 2.2.14. Transcriptome Sequencing Analysis

HepG2 cells treated with 60 μM TF3 and their untreated counterparts were collected, and total RNA was extracted using the TRIzol reagent. RNA samples that passed quality control were submitted to Servicebio (Wuhan Servicebio Biotechnology Co., Ltd., Wuhan, China) for transcriptome sequencing. Paired-end (PE150) sequencing was carried out on the DNBSEQ-T7RS platform (MGI Tech Co., Ltd., Shenzhen, China). Raw reads were first assessed for quality with FastQC v0.11.9. Low-quality sequences and adapters were removed with Trimmomatic v0.39 to obtain high-quality clean data. Subsequently, the cleaned reads were aligned to the reference genome via HISAT2 v2.1.0, followed by transcript assembly and quantification with StringTie v2.2.3. Finally, differential gene expression analysis was performed using DESeq2 v1.40.

#### 2.2.15. Statistical Analysis

All results were expressed as the mean ± S.D. of at least 3 independent experiments. One-way analysis of variance (ANOVA) was used for multiple comparisons followed by Dunnett’s test. The *p* values between 0.01 and 0.05 were regarded as significant difference, while *p* values less than 0.01 were regarded as highly significant differences. Data were visualized using Origin v2019 software.

## 3. Results

### 3.1. TF3 Targets Pin1 and Inhibits Its Activity

Molecular docking revealed that TF3 bound to the active site of Pin1 with a docking score of −8.9 kcal/mol, indicating a spontaneous interaction (Figure 1B). Furthermore, TF3 formed interactions with residues H59, L61, K63, W73, D112, C113, S114, S115, L122, G123, A124, Q129, M130, and S154. To further assess binding stability between Pin1 and TF3, molecular dynamics (MD) simulations were carried out for 100 ns with three independent replicates. As shown in Figure 1C,D, both RMSD and Rg trajectories remained largely flat, with no marked deviations, suggesting further that TF3 stably bound to the active site of Pin1.

As shown in Figure 1E, the purified Pin1 protein exhibited a molecular weight of approximately 21 kDa, and its purity met the requirements for subsequent experiments. As depicted in Figure 1F, TF3 exhibited an IC_50_ of 60.33 μM for PPIase activity of Pin1, aligning with previous reports [34]. Fluorescence titration experiments determined the binding constant (*K_a_*) of TF3 to Pin1 as 3.1 × 10^5^ mol/L, with a stoichiometric ratio (*n*) of 0.84 (Figure 1G). This indicates that TF3 exhibits high affinity for Pin1 and binds at a 1:1 stoichiometry to a single site. DARTS results showed that in HepG2 (Figure 1H and Figure S1), SK-Hep-1 (Figure 1I and Figure S1), and Huh-7 (Figure 1J and Figure S1) cells, Pin1 was resistant to pronase hydrolysis upon binding to TF3, further confirming the direct interaction between TF3 and Pin1 in these cells. In summary, the above findings suggest that TF3 binds to Pin1, implying that Pin1 may be one of its potential targets.

### 3.2. TF3 Inhibits Hepatocellular Carcinoma Cell Activity and Reduces the Expression Level of Pin1

**Figure 2:**
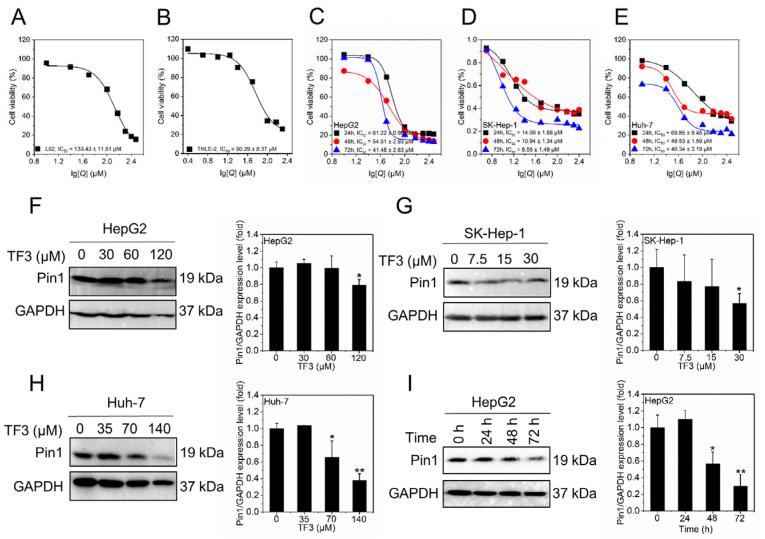
TF3 inhibits the viability of hepatocellular carcinoma cells and reduces the expression level of Pin1. (**A**,**B**). Cell viability of L02 and THLE-2 cells after 24 h of TF3 treatment. (**C**–**E**). Cell viability of HepG2, SK-Hep-1 and Huh-7 cells after treatment with TF3 for 24 h, 48 h, and 72 h. (**F**–**H**). Western blotting was used to detect the expression level of Pin1 in HepG2, SK-Hep-1 and Huh-7 cells after being treated with TF3 for 24 h. (**I**). Western blotting was used to detect the expression level of Pin1 in HepG2 cells treated with 60 μM TF3 at 0 h, 24 h, 48 h, and 72 h. * 0.01 < *p* < 0.05, significant difference, ** *p* < 0.01, highly significant difference.

THLE-2 and L02 are both normal hepatocytes that grow adherently and are commonly used in drug metabolism and toxicity studies. HepG2 (hepatoblastoma), Huh-7 (well-differentiated), and SK-Hep-1 (liver adenocarcinoma) are hepatocellular carcinoma cells characterized by plasma protein secretion, susceptibility to HCV, and high metastatic potential, respectively, making them suitable for research on mechanisms related to liver cancer. We first assessed the cytotoxic profile of TF3 on both normal and malignant liver cells. In two commonly used normal hepatocyte lines, L02 and THLE-2, the IC_50_ values at 24 h were 133.43 μM and 90.29 μM, respectively (Figure 2A,B). These results indicated that within a 24 h window and at concentrations below 90 μM, TF3 exhibited low toxicity toward normal hepatocytes, falling within a relatively safe dosage range. By contrast, TF3 markedly suppressed the viability of three hepatocellular carcinoma (HCC) lines. For HepG2 cells, the IC_50_ values at 24 h, 48 h, and 72 h were 61.22 μM, 54.51 μM, and 41.48 μM, respectively (Figure 2C). For SK-Hep-1 cells, the IC_50_ values were 14.09 μM, 10.94 μM, and 8.55 μM (Figure 2D). For Huh-7 cells, the IC_50_ values were 69.85 μM, 49.53 μM, and 40.34 μM (Figure 2E). Collectively, these results indicated that TF3 inhibits hepatocellular carcinoma cell viability in a dose-dependent and time-dependent manner.

Based on the IC_50_ values of TF3 for HepG2, SK-Hep-1, and Huh-7 cells, the low, medium, and high doses were established as follows: 30, 60, and 120 μM for HepG2; 7.5, 15, and 30 μM for SK-Hep-1; and 35, 70, and 140 μM for Huh-7 cells, respectively. We next investigated whether TF3 influences the expression of Pin1. In HepG2 (Figure 2F and Figure S2), SK-Hep-1 (Figure 2G and Figure S2), and Huh-7 (Figure 2H and Figure S2) cells, Pin1 expression levels progressively declined with increasing TF3 concentrations, suggesting that TF3 may bind to Pin1 and reduce its expression. Consistently, a time-course experiment in HepG2 cells showed a gradual decrease in Pin1 expression after 24 h, 48 h, and 72 h of TF3 exposure (Figure 2I and Figure S2), indicating that this reduction is time-dependent.

### 3.3. TF3 Induces Apoptosis in Hepatocellular Carcinoma Cells via the Mitochondrial Pathway

**Figure 3:**
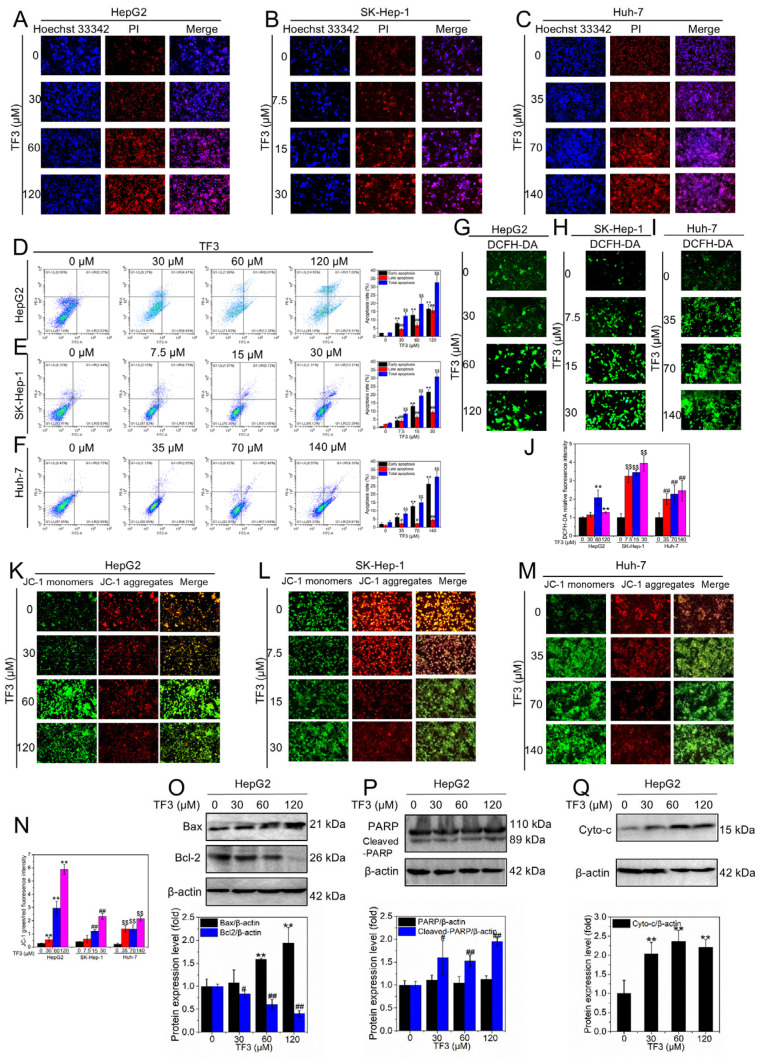
TF3 induces apoptosis in hepatocellular carcinoma cells through a mitochondrial-mediated pathway. (**A**–**C**). Detection of survival sand death of HepG2, SK-Hep-1 and Huh-7 cells after 24 h of treatment with different concentrations of TF3 using Hoechst 33342 and PI double staining. (**D**–**F**). Detection of apoptosis rate of HepG2, SK-Hep-1 and Huh-7 cells after 24 h of TF3 treatment using Annexin V-FITC/PI double staining combined with flow cytometry. (G–**I**). Detection of intracellular reactive oxygen species levels in HepG2, SK-Hep-1 and Huh-7 cells after 24 h of treatment with TF3 using the DCFH-DA fluorescent probe. (**J**). Quantitative analysis of intracellular ROS relative fluorescence intensity. (**K**–**M**). Detection of mitochondrial membrane potential changes in HepG2, SK-Hep-1 and Huh-7 cells after 24 h of treatment with TF3 using the JC-1 fluorescent probe. (**N**). Quantitative analysis of the red/green fluorescence ratio in JC-1 staining. (**O**). Western blotting was used to detect the protein expression levels of Bax and Bcl-2 in HepG2 cells after treatment with TF3 for 24 h. (**P**). Western blotting was used to detect the protein expression levels of PARP and cleaved-PARP in HepG2 cells after being treated with TF3 for 24 h. (**Q**). Western blotting was used to detect the protein expression level of cyto-c in HepG2 cells after treatment with TF3 for 24 h. 100× magnification, # 0.01 < *p* < 0.05, significant difference, **, ## and $$ *p* < 0.01, highly significant difference.

Hoechst 33342 readily penetrates the nuclear membrane, whereas propidium iodide (PI) is excluded from intact cells and enters only upon loss of membrane integrity. Therefore, normal cells and dead cells can be distinguished by double staining. As shown in Figure 3A–C, with the increase in TF3 concentration, the red fluorescence in HepG2 (Figure 3A), SK-Hep-1 (Figure 3B), and Huh-7 (Figure 3C) cells was enhanced, indicating that TF3 induces cell death in a dose-dependent manner. On the other hand, Annexin V-FITC can specifically bind to Phosphatidylserine (PS) that is everted in apoptotic cells, while PI can still only enter cells with lost membrane integrity. Therefore, this double staining system can be used to distinguish living cells, early apoptotic cells, late apoptotic cells and necrotic cells. As shown in Figure 3D–F, increasing concentrations of TF3 induced a dose-dependent increase in early, late, and total apoptosis rates in HepG2 (Figure 3D), SK-Hep-1 (Figure 3E), and Huh-7 (Figure 3F) cells, confirming its pro-apoptotic effect.

DCFH-DA is a membrane-permeable fluorescent probe that generates the highly fluorescent product DCF upon cellular uptake, with its fluorescence intensity reflecting intracellular ROS levels. Untreated HepG2 (Figure 3G), SK-Hep-1 (Figure 3H), and Huh-7 (Figure 3H) cells exhibited only faint green fluorescence, indicating low intracellular ROS levels. With increasing TF3 concentrations, green fluorescence intensity significantly enhanced in each cell line (Figure 3J), suggesting TF3 stimulates elevated intracellular ROS levels.

JC-1 accumulates within polarized mitochondria as red-fluorescent aggregates; upon mitochondrial depolarization, the dye disperses into green-fluorescent monomers. The red-to-green fluorescence ratio thus provides a sensitive readout of mitochondrial membrane potential. Untreated HepG2 (Figure 3K), SK-Hep-1 (Figure 3L), and Huh-7 (Figure 3M) cells predominantly exhibited red JC-1 aggregate fluorescence with weak green monomer fluorescence, indicating high mitochondrial membrane potential. With increasing TF3 concentrations, JC-1 staining showed a decrease in red fluorescence and a corresponding increase in green fluorescence (Figure 3N), indicating a reduction in mitochondrial membrane potential and suggesting that TF3 may induce apoptosis via the mitochondrial pathway.

Bax (pro-apoptotic protein) and Bcl-2 (anti-apoptotic protein) antagonistically regulate mitochondrial outer membrane permeability, with their ratio determining apoptosis initiation. As shown in Figure 3O and Figure S3, TF3 dose-dependently upregulated Bax while concomitantly downregulating Bcl-2 expression, indicating TF3 promotes hepatocellular carcinoma cell apoptosis. Apoptotic execution is characteristically accompanied by the specific cleavage of PARP, yielding signature fragments. As shown in Figure 3P and Figure S3, total PARP protein expression remained unchanged across different TF3 concentrations, while cleaved PARP expression significantly increased, further supporting the pro-apoptotic action of TF3. Additionally, cytochrome c (cyto-c) serves as a key signaling molecule in the mitochondrial apoptosis pathway. As shown in Figure 3Q and Figure S3, cytoplasmic cyto-c expression levels significantly increased with rising TF3 concentrations, confirming that TF3 primarily induces hepatocellular carcinoma apoptosis via the mitochondrial pathway.

### 3.4. TF3 Induces G_2_/M Phase Arrest in Hepatocellular Carcinoma Cells

**Figure 4:**
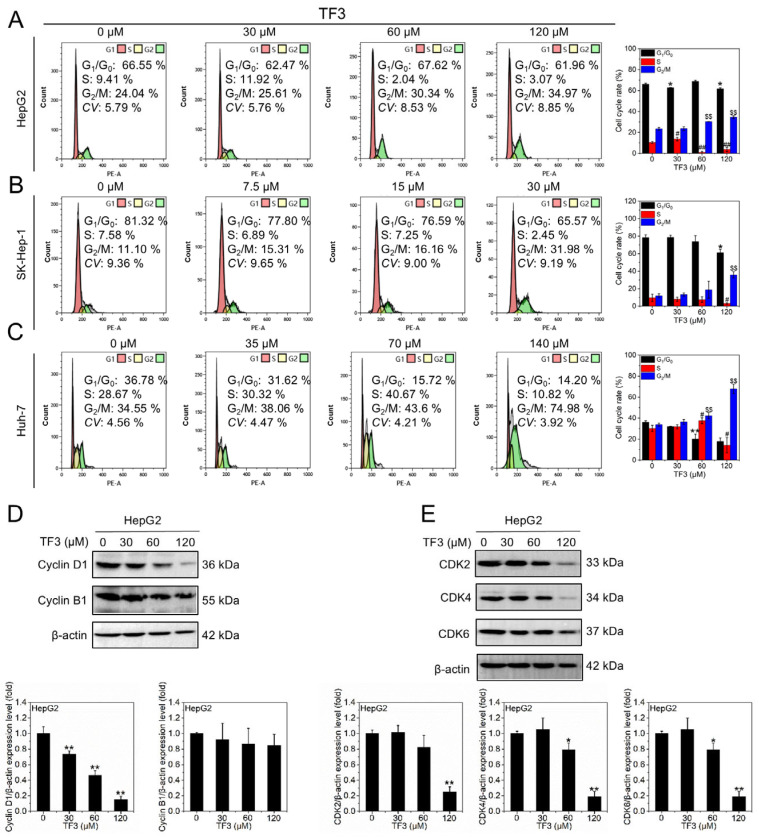
TF3 induces G_2_/M phase arrest in hepatocellular carcinoma cells. (**A**). Effect of TF3 treatment on HepG2 cell cycle distribution detected by PI staining. (**B**). Effect of TF3 treatment on cell cycle distribution of SK-Hep-1 cells detected by PI staining. (**C**). Effect of TF3 treatment on cell cycle distribution of Huh-7 cells detected by PI staining. (**D**). Western blotting was used to detect the protein expression levels of Cyclin D1 and Cyclin B1 in HepG2 cells after being treated with TF3 for 24 h. (**E**). Western blotting was used to detect the protein expression levels of CDK2, CDK4, and CDK6 in HepG2 cells after treatment with TF3 for 24 h. *, # 0.01 < *p* < 0.05, significant difference, **, ## and $$ *p* < 0.01, highly significant difference.

DNA content, reflected by PI fluorescence intensity, delineates the cell cycle phases: G_1_/G_0_ (diploid), S (DNA synthesis), and G_2_/M (tetraploid). As shown in Figure 4A–C, increasing TF3 concentrations progressively reduced the proportion of cells in the G_1_/G_0_ and S phases in HepG2 (Figure 4A), SK-Hep-1 (Figure 4B) and Huh-7 (Figure 4C) cells, while the proportion of cells in the G_2_/M phase gradually increased. These findings indicate that TF3 induces cell cycle arrest at the G_2_/M phase in hepatocellular carcinoma cells.

Consistent with this, Cyclin D1 expression levels significantly decreased with increasing TF3 concentrations, while Cyclin B1 expression remained unchanged (Figure 4D and Figure S4). Meanwhile, CDK2, CDK4, and CDK6 expression levels also significantly decreased with rising TF3 concentrations (Figure 4E and Figure S4). These data further confirmed that TF3 induces G_2_/M phase arrest in hepatocellular carcinoma cells.

### 3.5. TF3 Inhibits Proliferation, Invasion, and Migration of Hepatocellular Carcinoma Cells

**Figure 5:**
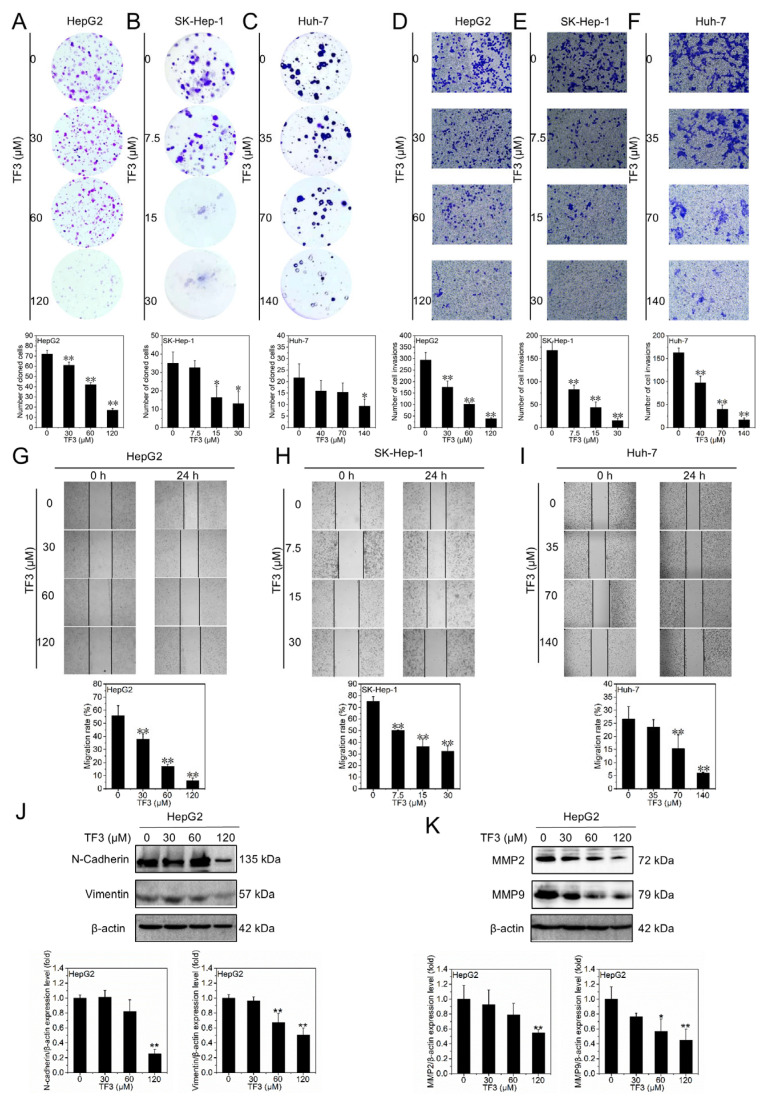
TF3 inhibits proliferation, invasion, and migration of hepatocellular carcinoma cells. (**A**–**C**). Colony formation assay to detect the colony-forming ability of HepG2, SK-Hep-1 and Huh-7 cells after treatment with TF3. (**D**–**F**). Transwell assay to detect the invasive ability of HepG2, SK-Hep-1 and Huh-7 cells after treatment with TF3. (**G**–**I**). Scratch wound healing assay to detect the migration ability of HepG2, SK-Hep-1 and Huh-7 cells after treatment with TF3. (**J**). Western blotting was used to detect the protein expression levels of N-Cadherin and Vimentin in HepG2 cells after treatment with TF3 for 24 h. (**K**). Western blotting was used to detect the protein expression levels of MMP2 and MMP9 in HepG2 cells after treatment with TF3 for 24 h. 100× magnification, * 0.01 < *p* < 0.05, significant difference, ** *p* < 0.01, highly significant difference.

The colony formation assay assesses the in vitro proliferative and colony-forming capacity of individual cells. As shown in Figure 5A–C, colony formation by HepG2 (Figure 5A), SK-Hep-1 (Figure 5B), and Huh-7 (Figure 5C) cells decreased in a dose-dependent manner with increasing TF3 concentrations, indicating that TF3 inhibits hepatocellular carcinoma cell proliferation.

Transwell assay quantitatively analyzes cell invasion capacity. As shown in Figure 5D–F, increasing concentrations of TF3 reduced the number of cells migrating through the matrix in HepG2 (Figure 5D), SK-Hep-1 (Figure 5E), and Huh-7 (Figure 5F) cells, indicating that TF3 inhibits the invasion of hepatocellular carcinoma cells.

Scratch wound healing assays are commonly used to assess cell migration capacity. As shown in Figure 5G–I, treatment with TF3 significantly reduced the wound healing area of HepG2 (Figure 5G), SK-Hep-1 (Figure 5H), and Huh-7 (Figure 5I) cells in a concentration-dependent manner, indicating that TF3 inhibits the migration of hepatocellular carcinoma cells.

N-Cadherin and Vimentin typically promote cell migration and invasion, while matrix metalloproteinases MMP2 and MMP9 synergistically enhance this process by degrading the extracellular matrix. As shown in Figure 5J,K and Figure S5, the expression levels of N-Cadherin, Vimentin, MMP2, and MMP9 all decreased significantly with increasing TF3 concentrations, further confirming that TF3 inhibits the migration and invasion capabilities of hepatocellular carcinoma cells.

### 3.6. TF3 Inhibits MAPK and PI3K/AKT Signaling Pathways

**Figure 6:**
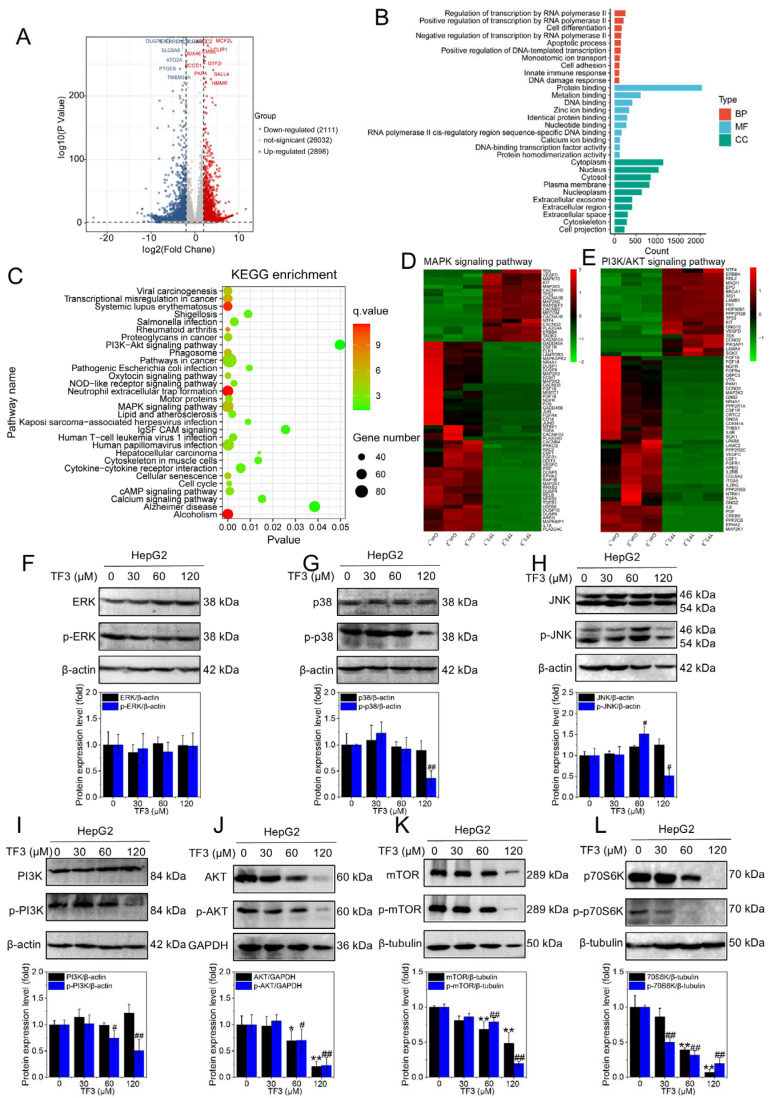
TF3 blocks MAPK and PI3K/AKT signaling pathways. (**A**). Volcano plot of differentially expressed genes in HepG2 cells after TF3 treatment. (**B**). GO enrichment analysis of differentially expressed genes in HepG2 cells after TF3 treatment. (**C**). KEGG enrichment analysis of differentially expressed genes in HepG2 cells after TF3 treatment. (**D**). Heatmap of differentially expressed genes in the MAPK signaling pathway. (**E**). Heatmap of differentially expressed genes in the PI3K/AKT signaling pathway. (**F**). Western blotting was used to detect the protein expression levels of ERK and p-ERK in HepG2 cells after treatment with TF3 for 24 h. (**G**). Western blotting was used to detect the protein expression levels of p38 and p-p38 in HepG2 cells after being treated with TF3 for 24 h. (**H**). Western blotting was used to detect the protein expression levels of JNK and p-JNK in HepG2 cells after being treated with TF3 for 24 h. (**I**). Western blotting was used to detect the protein expression levels of PI3K and p-PI3K in HepG2 cells treated with TF3 for 24 h. (**J**). Western blotting was used to detect the protein expression levels of AKT and p-AKT in HepG2 cells after treatment with TF3 for 24 h. (**K**). Western blotting was used to detect the protein expression levels of mTOR and p-mTOR in HepG2 cells after being treated with TF3 for 24 h. (**L**). Western blotting was used to detect the protein expression levels of p70S6K and p-p70S6K in HepG2 cells after being treated with TF3 for 24 h. * and # 0.01 < *p* < 0.05, significant difference, ** and ## *p* < 0.01, highly significant difference.

The volcano chart visually presents the results of the difference analysis through a scattered distribution. As shown in Figure 6A, with a threshold of ≥2, after 60 μM TF3 treatment of HepG2 cells, a total of 5009 differentially expressed genes were screened out, among which 2898 genes were up-regulated and 2111 genes were down-regulated. As shown in Figure 6B, GO enrichment analysis of the differentially expressed genes was performed, covering three categories: biological processes (BP), molecular functions (MF), and cellular components (CC). In the BP category, TF3 treatment significantly enriched in apoptosis-related pathways, consistent with previous experimental findings, further suggesting that TF3 induces apoptosis in hepatocellular carcinoma cells.

KEGG pathway enrichment (Figure 6C) revealed that TF3-treated HepG2 cells exhibited significant enrichment in genes associated with the cell cycle, MAPK, and PI3K/AKT signaling pathways. To better resolve how TF3 interfaces with these signaling networks, we generated heatmaps centered on DEGs belonging to the MAPK and PI3K/AKT families. As shown in Figure 6D, TF3 extensively remodeled the MAPK transcriptional landscape, with 50 genes downregulated and 19 upregulated. A similar pattern emerged for the PI3K/AKT axis, with 40 genes downregulated and 20 upregulated (Figure 6E).

The three principal arms of the MAPK pathway (ERK, JNK, and p38) are classically triggered by growth factors, stress, and inflammatory stimuli, respectively, and together orchestrate proliferation, differentiation, stress adaptation, and apoptosis. As shown in Figure 6F and Figure S6, neither the expression of ERK nor its phosphorylated form p-ERK exhibited significant changes in TF3-treated HepG2 cells. The results in Figure 6G and Figure S6 show that the total protein expression level of p38 does not change significantly. However, under the treatment of high-concentration TF3, the expression of its phosphorylated form p-p38 is significantly down-regulated. This phenomenon may be related to the inhibitory effect of TF3 on Pin1. Figure 6H and Figure S6 indicates that the total protein expression of JNK remains stable, while the expression level of its phosphorylated form p-JNK showed a trend of first increasing and then decreasing, suggesting that the JNK MAPK pathway may be instantaneously activated during TF3 treatment.

PI3K/AKT constitutes a classic signal transduction pathway: After PI3K is activated, it phosphorylates AKT, which in turn activates mTOR. mTOR further phosphorylates its downstream effector protein p70S6K, cooperatively regulating cell growth, proliferation and metabolism. The results in Figure 6I–L and Figure S6 show that in TF3-treated HepG2 cells, the expression levels of PI3K, AKT, mTOR and their phosphorylated forms (p-PI3K, p-AKT, p-mTOR), as well as the downstream effector protein p70S6K and its phosphorylated form (p-p70S6K), were significantly decreased. The above results indicate that TF3 can inhibit the activation of the PI3K/AKT signaling pathway.

## 4. Discussion

Pin1 is highly expressed in various malignant tumors [46,47]. It stabilizes its active conformation by isomerizing the phosphorylated serine/threonine-proline motif substrate protein, thereby abnormally activating multiple cancer-promoting signaling pathways [37,48]. It has thus become a potential target for anti-tumor drugs [49]. This study found that TF3 can bind to the active center of Pin1 with high affinity (*K_a_* = 3.1 × 10^5^ mol/L) and effectively inhibit its PPIase enzyme activity (IC_50_ = 60.33 μM), suggesting that Pin1 may be one of the potential targets of TF3. Particularly importantly, the binding of TF3 to Pin1 can induce a dose-dependent and time-dependent decrease in the intracellular Pin1 protein level. Compared with some reported Pin1 inhibitors (such as epigallocatechin-3-gallate, 6,7,4′-trihydroxyisoflavone, VS-10), TF3 also has the characteristic of reducing the expression of Pin1 after binding, suggesting that it may have a stronger ability to interfere with signaling pathways and anti-tumor potential [32,50,51].

At the cellular phenotypic level, TF3 exhibited significant growth inhibitory effects on various hepatoma cell lines (HepG2, SK-Hep-1, Huh-7), and its half-maximal inhibitory concentration (IC_50_) on cancer cell viability was significantly lower than the toxic dose for normal liver cells, indicating that it has good tumor cell selectivity. In addition, TF3 treatment can induce apoptosis through the mitochondrial pathway, manifested as elevated reactive oxygen species (ROS) levels, decreased mitochondrial membrane potential, cytochrome c release, upregulation of the Bax/Bcl-2 ratio, and increased PARP cleavage. Meanwhile, TF3 arrested the cell cycle at the G_2_/M phase, accompanied by the downregulation of the expression of cell cycle positive regulatory proteins Cyclin D1 and CDK2/4/6. Furthermore, TF3 significantly inhibits the clone formation, invasion and migration abilities of liver cancer cells, which is consistent with the down-regulation of the expression of migration-related markers N-Cadherin, Vimentin, and matrix metalloproteinases MMP2 and MMP9. The above anti-tumor phenotypic experimental results are highly consistent with the known functions of Pin1 in regulating core biological processes such as cell cycle, apoptosis, proliferation and migration [51,52], further confirming that TF3 exerts anti-liver cancer effects by targeting and inhibiting Pin1.

Transcriptomic analysis further focused the mechanism of action of TF3 on two classical signaling pathways, MAPK and PI3K/AKT, both of which are important nodes in the Pin1 regulatory network [52,53]. In the MAPK pathway, TF3 treatment significantly inhibited the phosphorylation level of p38, while the phosphorylation of JNK showed a dynamic change of first increasing and then decreasing. The down-regulation of p38 activity may be related to the stability changes of substrate proteins (such as phosphatases or kinases that regulate p38 activity) mediated by Pin1 inactivation. The activation of the JNK signal may be an early stress response, which gradually decays with the continuous inhibition of Pin1 function. Particularly importantly, TF3 exhibited a comprehensive inhibitory effect on the core pathway of PI3K/AKT, which regulates cell growth and metabolism. The total protein and phosphorylation levels at key nodes of this pathway consistently decreased. Previous studies have confirmed that Pin1 can positively regulate the PI3K/AKT signaling by isomerizing multiple pathway components such as AKT [54]. Therefore, the inhibition of Pin1 by TF3 may disrupt the positive regulatory network of this pathway, thereby deeply blocking its signal transduction. The above-mentioned mechanisms constitute the core molecular basis for phenotypes such as cell cycle arrest, apoptosis and proliferation inhibition induced by TF3.

Despite these promising findings, several limitations should be acknowledged. First, the absence of in vivo validation, as well as the lack of experiments using primary HCC samples, means that the anti-tumor efficacy of TF3 remains to be confirmed in more clinically relevant models. Second, although TF3 shows some selectivity for liver cancer cells over normal hepatocytes (L02 and THLE-2), the narrow selectivity window raises concerns about potential off-target toxicity in normal tissues. Third, at the concentrations used, the large number of differentially expressed genes suggests possible non-specific pathway inhibition beyond on-target effects. Whether the observed modulation of MAPK and PI3K/AKT signaling is entirely mediated by Pin1 inhibition or partly due to non-specific interactions requires further investigation. Fourth, the potential combination therapy of TF3 with sorafenib or lenvatinib, as well as the possible regulation of PD-L1 by TF3 through Pin1 inhibition, represent important issues that warrant further investigation in future studies.

**Figure 7:**
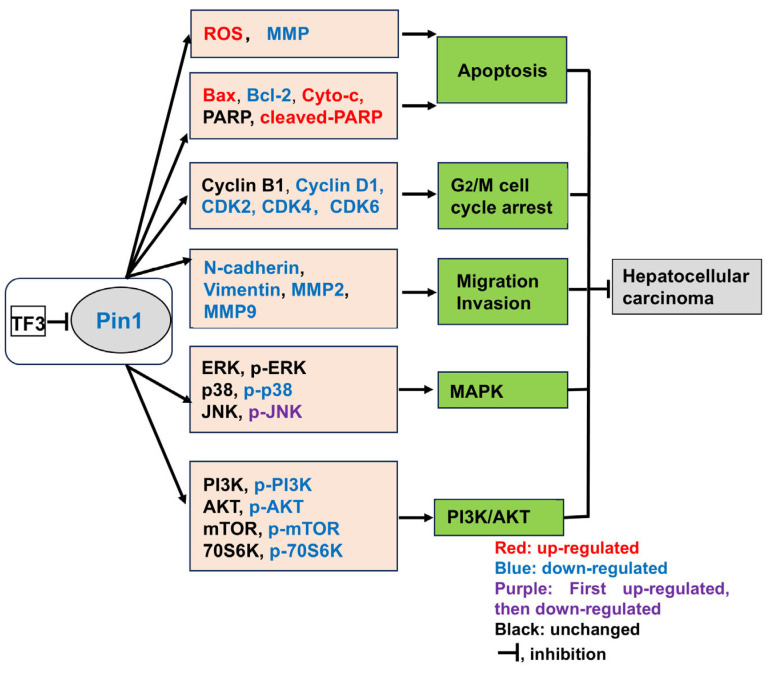
Hypothetical diagram of the potential mechanism of action of TF3 targeting Pin1 to inhibit hepatocellular carcinoma.

In summary, this study has for the first time clarified that TF3 is a novel Pin1-targeted inhibitor. It binds to Pin1 with high affinity and reduces its expression, thereby dually inhibiting the activities of the MAPK and PI3K/AKT signaling pathways, and ultimately triggering multi-dimensional anti-tumor effects in liver cancer cells (Figure 7). This study not only expands the pharmacological mechanisms of action of theaflavin compounds, but also provides promising lead compounds for the development of liver cancer treatment drugs targeting Pin1, an important target.

## 5. Conclusions

This study confirmed that TF3 is an effective inhibitor of peptidyl cis-trans isomerase Pin1. TF3 can bind to the active center of Pin1 with high affinity and inhibit its enzymatic activity, reducing its expression within cells. This targeting effect mediates the extensive anti-liver cancer activity of TF3, which is achieved by inducing apoptosis through the mitochondrial pathway, triggering G_2_/M phase cell cycle arrest, and inhibiting cell proliferation, migration and invasion. Transcriptomics and protein-level analysis indicated that the anti-tumor effect of TF3 was closely related to the regulation of the MAPK and PI3K/AKT signaling pathways. At the protein level, TF3 downregulates phospho-p38 and exerts dynamic control over JNK phosphorylation; at the same time, it markedly blunts activation along the PI3K/AKT pathway. In summary, this study demonstrates that TF3 targets Pin1 to synergistically inhibit both the MAPK and PI3K/AKT signaling networks, representing the core mechanism underlying its anti-hepatocellular carcinoma activity.

## Data Availability

The original contributions presented in this study are included in the article/Supplementary Material. Further inquiries can be directed to the corresponding author.

## Supplementary Materials

The following supporting information can be downloaded at: https://www.mdpi.com/article/10.3390/biom16040583/s1, Figure S1: Uncropped images of full Western blots of Figure 1; Figure S2: Uncropped images of full Western blots of Figure 2; Figure S3: Uncropped images of full Western blots of Figure 3; Figure S4: Uncropped images of full Western blots of Figure 4; Figure S5: Uncropped images of full Western blots of Figure 5; Figure S6: Uncropped images of full Western blots of Figure 6.
