# Supplementary material for: Theaflavin-3,3′-Digallate Targets Pin1 to Suppress Hepatocellular Carcinoma Malignant Proliferation Through Modulation of MAPK and PI3K/AKT Signaling Pathways In Vitro
Source: Biomolecules. 2026 Apr 14;16(4):583. doi: 10.3390/biom16040583 (PMC13113847; doi:10.3390/biom16040583)
Supplement: Supplementary file 1 [file biomolecules-16-00583-s001.zip › biomolecules-4215203-supplementary.pdf]

Supplementary materials

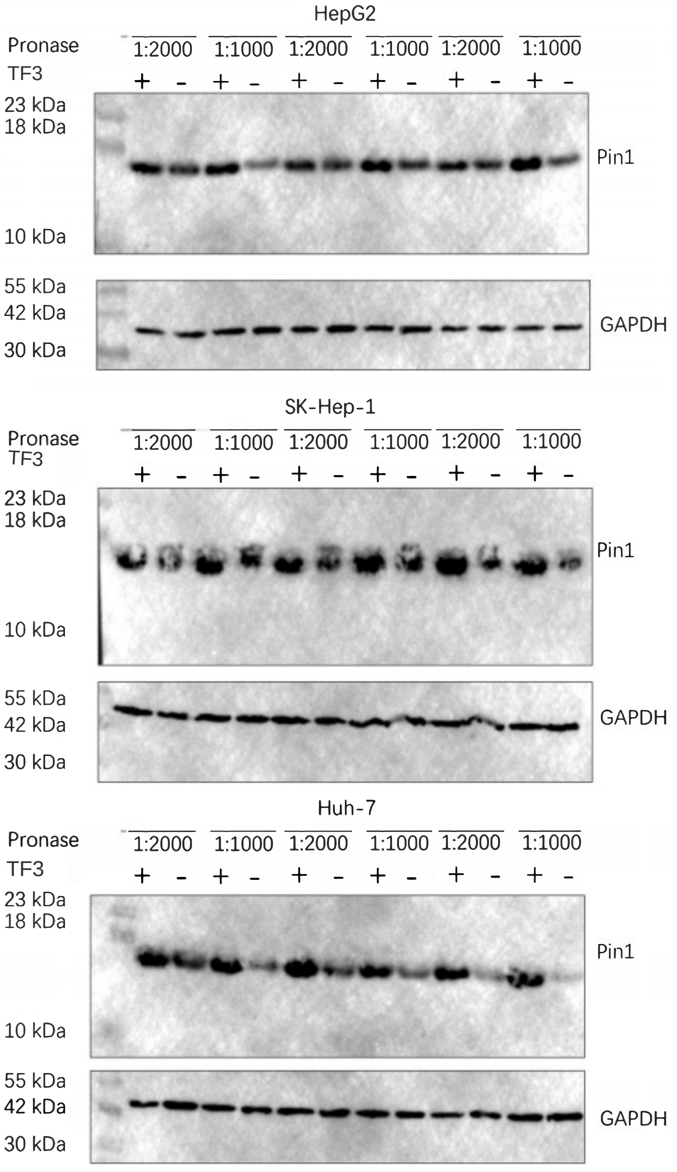

Figure S1. Uncropped images of full Western blots of Figure 1.

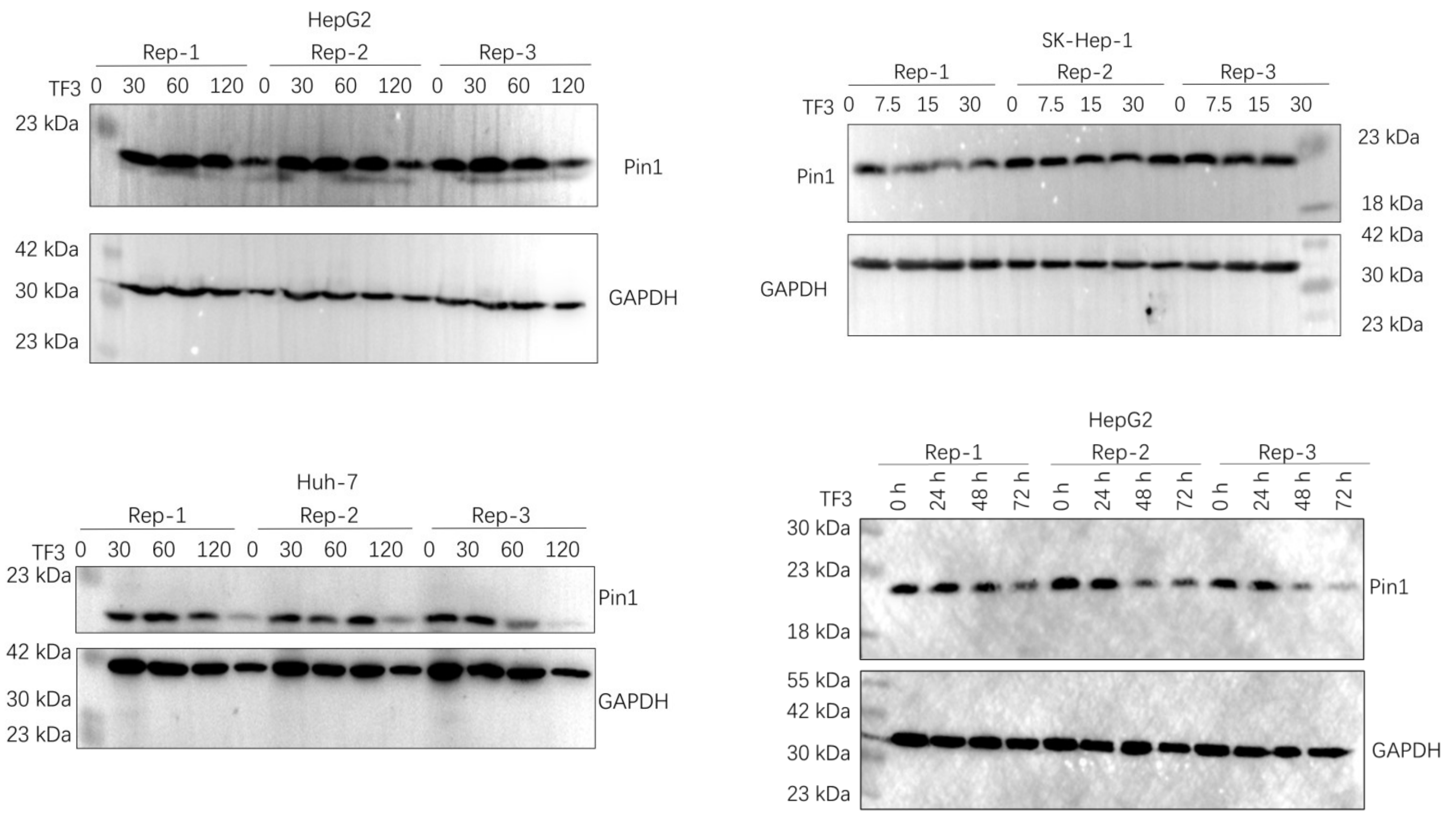

Figure S2. Uncropped images of full Western blots of Figure 2.

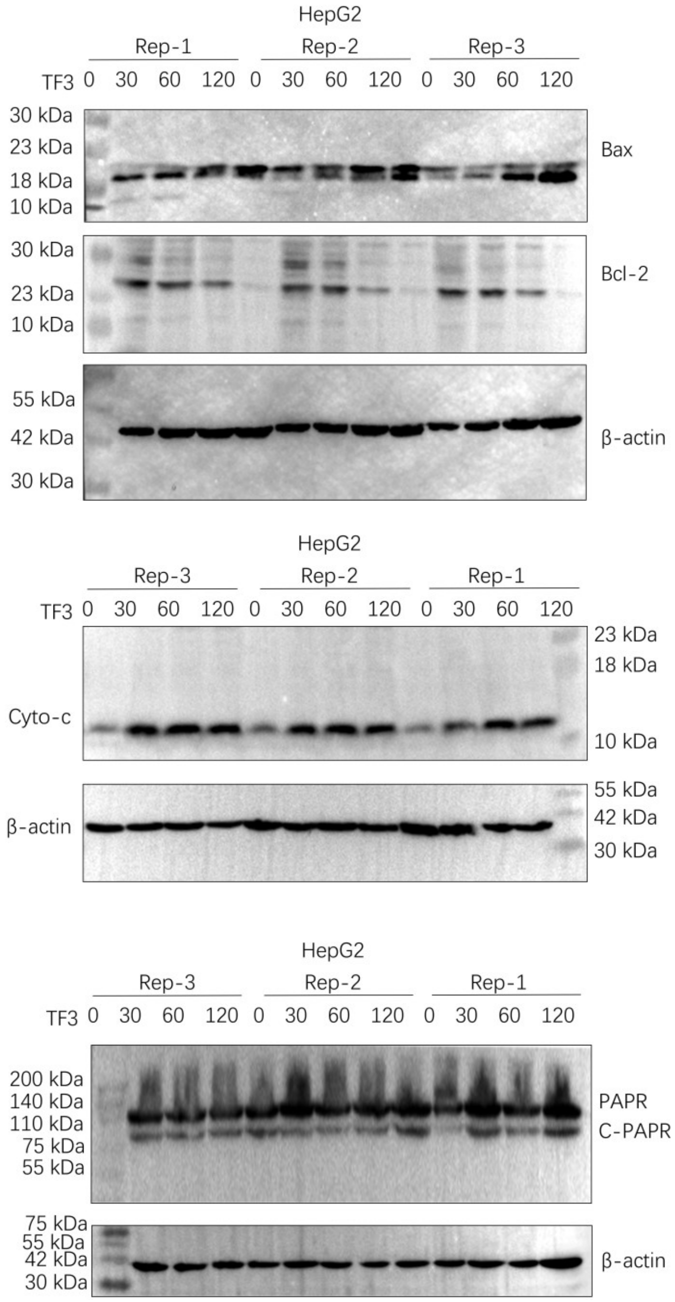

Figure S3. Uncropped images of full Western blots of Figure 3.

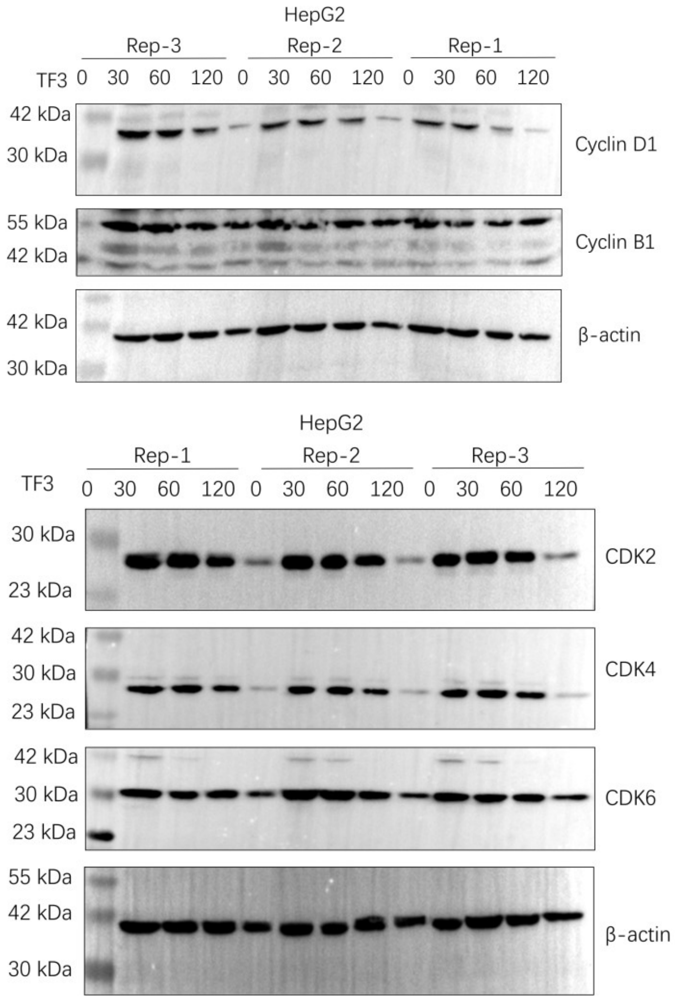

Figure S4. Uncropped images of full Western blots of Figure 4.

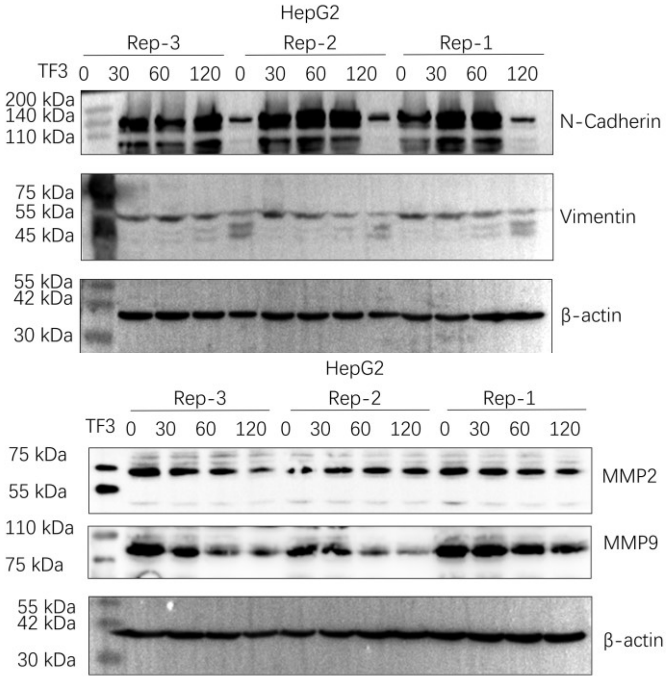

Figure S5. Uncropped images of full Western blots of Figure 5.

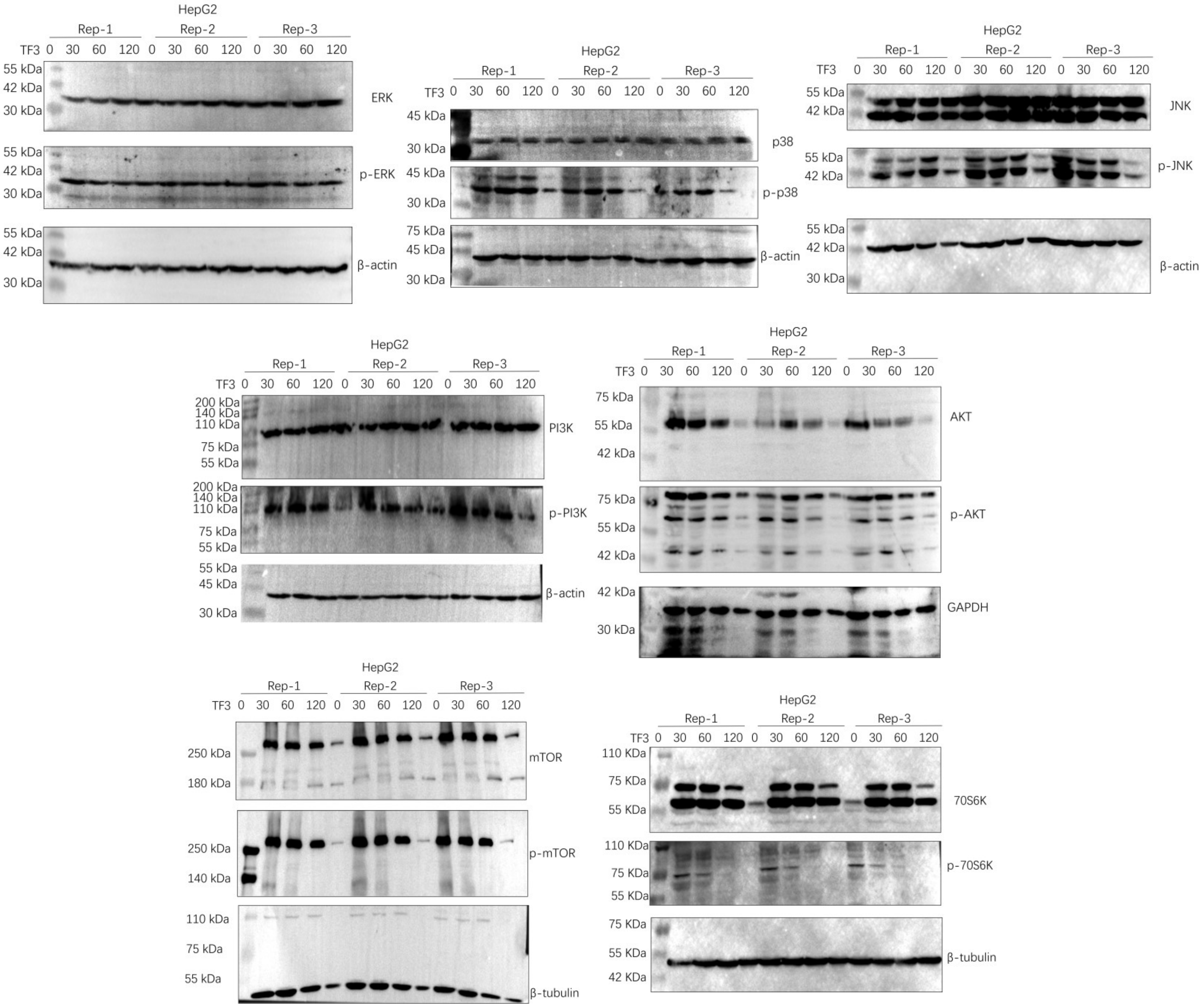

Figure S6. Uncropped images of full Western blots of Figure 6.
